# Comparison of tidal volume and airway pressure during neonatal resuscitation in mask leaks

**DOI:** 10.1111/ped.70139

**Published:** 2025-07-02

**Authors:** Fumihiko Takatori, Tetsuya Isayama, Shigeharu Hosono, Kiyotaka Iwasaki

**Affiliations:** ^1^ Cooperative Major in Advanced Biomedical Sciences Joint Graduate School of Tokyo Women's Medical University and Waseda University, Waseda University Tokyo Japan; ^2^ Ventilator & Anesthesia Products Department Nihon Kohden Corporation Tokyo Japan; ^3^ Division of Neonatology National Center for Child Health and Development Tokyo Japan; ^4^ Division of Neonatology Jichi Medical University Saitama Medical Center Saitama Japan; ^5^ Department of Modern Mechanical Engineering School of Creative Science and Engineering, Waseda University Tokyo Japan; ^6^ Department of Integrative Bioscience and Biomedical Engineering Graduate School of Advanced Science and Engineering, Waseda University Tokyo Japan; ^7^ Institute for Medical Regulatory Science Waseda University Tokyo Japan

**Keywords:** infant newborn, positive‐pressure respiration, resuscitation

## Abstract

**Background:**

Providing positive‐pressure ventilation (PPV) to the lungs is essential for neonatal resuscitation. Accurate PPV requires a precise measurement of tidal volume and airway pressure, with airway obstruction and mask leakage being the primary concerns for ineffective ventilation. This study aimed to investigate the differences between tidal volume and airway pressure measured by a respiratory function monitor (RFM) and the actual values delivered to the lungs in scenarios involving mask leaks, using a system comprising a PPV device, a face mask, and an artificial lung model.

**Methods:**

Three experiments were conducted to assess mask leakage (1) under varying lung conditions, (2) under different ventilation rates, and (3) using different PPV devices. Two RFMs were used, one in the test lung and the other in the mask. Trends in those data were assessed by means of a correlation graph.

**Results:**

Mask leakage resulted in an underestimation of the actual tidal volume, with the effect intensifying as the leak percentage increased. PPV devices using a compressed gas source demonstrated less reduction in lung tidal volume (from 15 to 12 mL) owing to mask leaks compared with those without such a source (from 16 to 5 mL).

**Conclusions:**

Significant discrepancies were observed between RFM readings and test lung values for tidal volume. These findings highlight the importance of accurate monitoring to prevent lung injury caused by excessive tidal volume, particularly in the presence of mask leaks. Accurately measuring tidal volume in the presence of mask leaks presents a significant challenge for the future.

## INTRODUCTION

Approximately 15% of newborns do not initiate spontaneous breathing at birth, and 5% require positive‐pressure ventilation (PPV).^1^, ^2^ With an estimated 140 million births worldwide each year, approximately 7 million infants require PPV to initiate their first breath.^3^

To perform PPV safely and effectively, delivering adequate tidal volume and airway pressure to the lungs is essential. The primary causes of ineffective mask ventilation are airway obstruction and mask leakage.^4^, ^5^ Recently, a respiratory function monitor (RFM) has been commercialized, enabling the real‐time display of airway pressure and tidal volume. The use of RFM helped reduce obstruction or mask leaks^6^ and has been associated with a decrease in intracerebral hemorrhage.^7^ However, these parameters were measured at the flow sensor between the mask and the PPV device, rather than directly in the patient's lungs. Monitor readings were not intended to align with lung measurements, and this measurement error becomes particularly significant in cases of obstruction or mask leakage.

Schmölzer et al. defined a 75% reduction in expiratory tidal volume and a mask leak of >75% as clinically significant.^4^ However, a standardized understanding of these quantitative values has yet to be established.^3^ Additionally, O'Donnell examined the volume at the mask and test lung using a T‐piece (TP) resuscitator device that required a compressed gas source and found that mask leaks tended to underestimate the tidal volume. However, the mask leak was not quantitatively controlled, and the study was limited to the use of the TP resuscitator.^8^

The calculation of mask leak involves dividing the difference between the inspiratory and expiratory tidal volumes by the inspiratory tidal volume. Owing to the gap between the mask and the patient's face, accurately determining the exact tidal volume is not possible. The extent of mask leakage is thought to significantly affect the adequate tidal volume delivered to the patient's lungs. Additionally, the differences in airway pressure must be examined. Thus, we hypothesized that an increase in mask leakage could lead to variations in tidal volume or airway pressure between the sensor on the mask and the lungs. Consequently, this study aimed to investigate the tidal volume and airway pressure displayed by the RFM and compare these values with those entering the lungs by intentionally inducing mask leaks using an experimental system comprising a PPV device, a face mask, and an artificial lung model.

## METHODS

Three experiments were conducted in this study: (1) mask leakage under varying test lung conditions, (2) mask leakage at different ventilation rates, and (3) mask leakage using different PPV devices.

### Test setting

**FIGURE 1:**
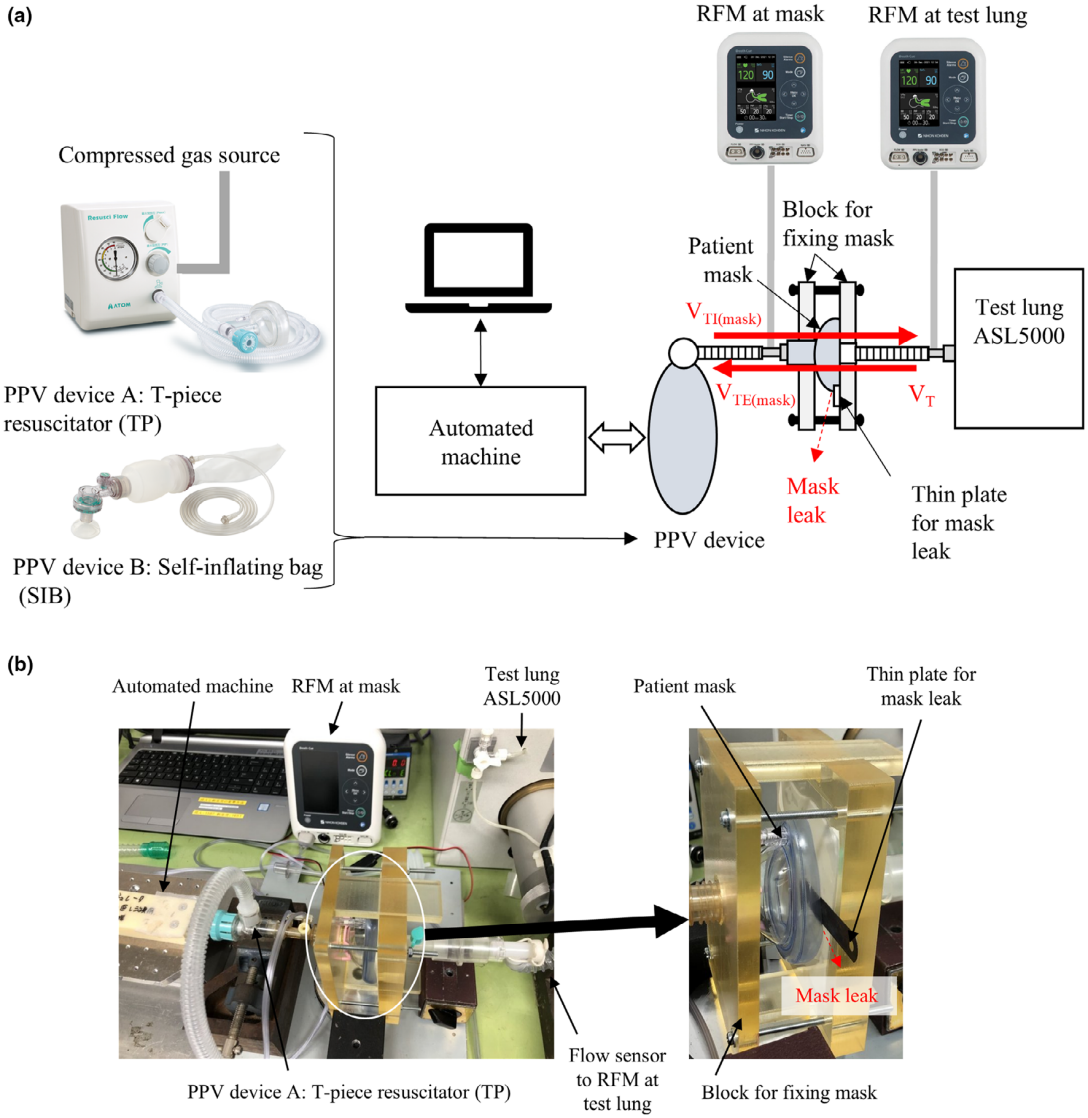
(a) A schematic diagram of the experimental environment. RFM (shown on the right) monitors tidal volume and airway pressure at the test lung. RFM (shown on the left) monitors tidal volume and airway pressure at the mask. The automated machine replicates the PPV technique. Thin plate was placed between the mask and the block. (b) A picture of the experimental environment.

This study used two RFMs (Breath‐Cue NRM‐1300; Nihon Kohden, Tokyo, JP). One RFM was positioned between the test lung and the face mask, whereas the other was placed between the PPV device and the face mask (Figure 1a,b). The RFM is capable of assessing expiratory tidal volume (V_TE_), ventilation rate (VR), peak inspiratory pressure (PIP), positive end‐expiratory pressure (PEEP), and mask leaks for each breath. Mask leak was calculated by comparing the inspiratory tidal volume (V_TI(mask)_) and the expiratory tidal volume (V_TE(mask)_). Mask leakage was expressed as %leak = (V_TI(mask)_ − V_TE(mask)_)/V_TI(mask)_ × 100 [%].^8^

PIP_(mask)_, PEEP_(mask)_, V_TE(mask)_, and V_TI(mask)_ were measured at the PPV device and the face mask. By contrast, PIP, PEEP, and V_TE_ without the (mask) designation refer to the pressures or volumes entering the test lung.

An automated machine, designed to replicate the PPV procedure, was utilized to compress the bag with movements up to ±300 mm/s. This was achieved using a linear motor (AZM66AC Stepping Motor, Oriental Moter, Tokyo, JP) and controller (NI cDAQ‐9174, National Instruments, Texas, USA). The speed and distance were adjusted to achieve a target PIP and PEEP under 0% leaks. A patient mask (air cushion mask size 1, Atom Medical, Tokyo, JP) was securely attached using two blocks to ensure a leak‐free connection and then connected to the test lung model. Respiratory distress syndrome (RDS) lung (compliance: 0.8 mL/cmH_2_O, resistance: 105 cmH_2_O/L/s) and normal lung (compliance: 3 mL/cmH_2_O, resistance: 38 cmH_2_O/L/s) were simulated using a test lung (ASL‐5000, IngMar Medical, Pennsylvania, US).^9^

Two PPV devices were used as follows: TP resuscitator (Resusci Flow, Atom Medical, Tokyo, JP) and a self‐inflating bag (SIB, MMI resuscitator NVY280mL, Muranaka Medical Instruments, Osaka, JP) The TP resuscitator was set at a fixed gas flow rate of 10 L/min. Although some SIBs can be fitted with PEEP valves,^10^ an SIB without a PEEP valve was used based on previous literature showing a relationship between leakage and PEEP. This configuration was assumed to be more likely to highlight the differences in ventilation volume. In addition, the SIB was not connected to a gas source. Flow‐inflating bags (FIBs), commonly used in clinical practice, were excluded due to their wide variation in PIP and PEEP values.^11^ Both FIB and TP rely on an external positive‐pressure source, leading to the decision to exclude FIB from the study. The inspiratory to expiratory (I: E) ratio was set at 1:2, whereas the ventilation rate was set at 50 breaths/min.

### Experiment

#### Mask leak under different test lung conditions

The RDS and normal lung models were compared. The PIP/PEEP was set at 20/5 cmH_2_O for RDS and 10/5 cmH_2_O for normal lungs. To simulate a quantifiable mask leak, a thin plate was placed between the mask and the block (Figure 1b). The plate thickness could be adjusted to simulate a large mask leak. When the leak exceeded 100%, the V_TE_ could no longer be recognized as breath by the RFM; therefore, the %leak was adjusted between 0% and 99%. Despite increasing leakage, the speed and depth of manual ventilation remained constant. The PPV device used a TP with the VR set at 50 breaths/min.

#### Mask leak under different ventilation rates

The VR was set to an upper limit of 40 breaths per minute and a lower limit of 60 breaths per minute, as recommended by the guidelines.^3^ The test lungs were adjusted to simulate RDS with PIP and PEEP of 20/5 cmH_2_O at 0% leak, and the mask leak gradually increased. The PPV device used was a TP, with the VR set at 50 breaths/min.

#### Mask leak in different PPV devices

SIBs and TPs were used for comparison, as both are commonly used in clinical practice. The VR was set at 50 breaths per minute, and the test lungs were adjusted to simulate RDS, with PIP and PEEP of 20/5 cmH_2_O at 0% leak. Subsequently, the percentage of mask leaks gradually increased.

### Statistical analysis

Experiments were conducted using a simulator to ensure data reproducibility. We used Pearson's correlation to analyze the relationship between %leak and tidal volume or airway pressure, visualized with a correlation graph. The data were averaged three times after adjusting the set point and stabilized after a few breaths. Confidence intervals are not shown when all three datasets are identical. The RFM measurement accuracy was ±5% of the measured value or 2 mL, with any differences beyond that attributed to the measurement environment. The graph shapes and trends are discussed. Pearson's correlations and graphs were constructed using Microsoft Excel (Microsoft 365 MSO, version 2501).

## RESULTS

### Mask leak under different test lung conditions

**FIGURE 2:**
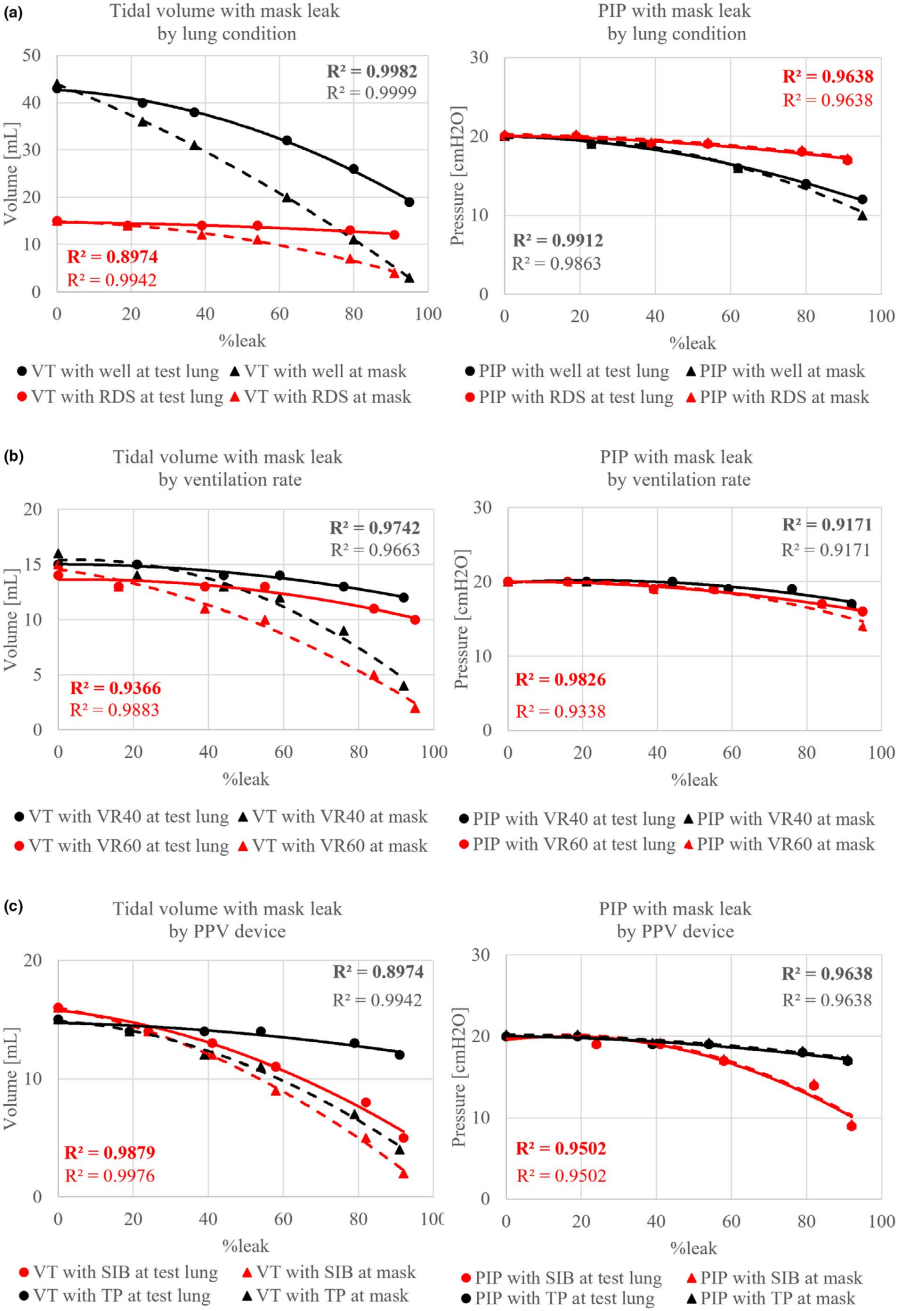
(a) Tidal volume and peak inspiratory pressure results at different mask leaks by lung condition. VTE delivered to the normal lung was 44 mL, whereas it was only 15 mL for the RDS lung when there was a 0%leak. The tidal volume measured at the mask and test lung began to diverge as the percentage of mask leakage increased. *R* means Pearson's correlation, and bold text indicates measured at test lungs, and thin text indicates measured at mask. (b) Tidal volume and peak inspiratory pressure results at different mask leaks by ventilation rate. As the mask leakage increased, both tidal volume and airway pressure decreased. This was particularly noticeable at higher ventilation rates and with more significant differences. *R* means Pearson's correlation, and bold text indicates measured at test lungs, and thin text indicates measured at mask. (c) Tidal volume and peak inspiratory pressure results at different mask leaks by PPV devices. T‐piece resuscitator did not reduce tidal volume as much as the SIB as mask leaks increased. The SIB showed a more significant decrease in airway pressure than the T‐piece resuscitator with increasing mask leaks. *R* means Pearson's correlation, and bold text indicates measured at test lungs, and thin text indicates measured at mask.

When the airway pressure was set to the same level for both the normal lung and RDS lung, the V_TE_ delivered to the normal lung was 44 mL, whereas only 15 mL was delivered to the RDS lung in the absence of mask leakage (0% leak). Both airway pressure and tidal volume measured at the mask and test lung decreased as the percentage of mask leakage increased. The tidal volume measured at the mask and test lungs began to diverge as the percentage of mask leakage increased (Figure 2a). As the mask leakage increased, the normal lungs experienced a more significant decrease in tidal volume and airway pressure compared with the RDS lungs. Airway pressure remained nearly constant in the mask and test lungs, regardless of the percentage of mask leakage.

### Mask leak under different ventilation rates

As the percentage of mask leakage increased, both tidal volume and airway pressure decreased. This effect was particularly pronounced at higher ventilation rates, with more significant differences observed (Figure 2b).

### Mask leak in different PPV devices

When different PPV devices were compared, the TP resuscitator caused a slight reduction in tidal volume, although not as pronounced as the decrease observed with the SIB as mask leakage increased. Similarly, the SIB demonstrated a more significant decrease in airway pressure compared with the TP resuscitator as mask leakage increased (Figure 2c).

**FIGURE 3:**
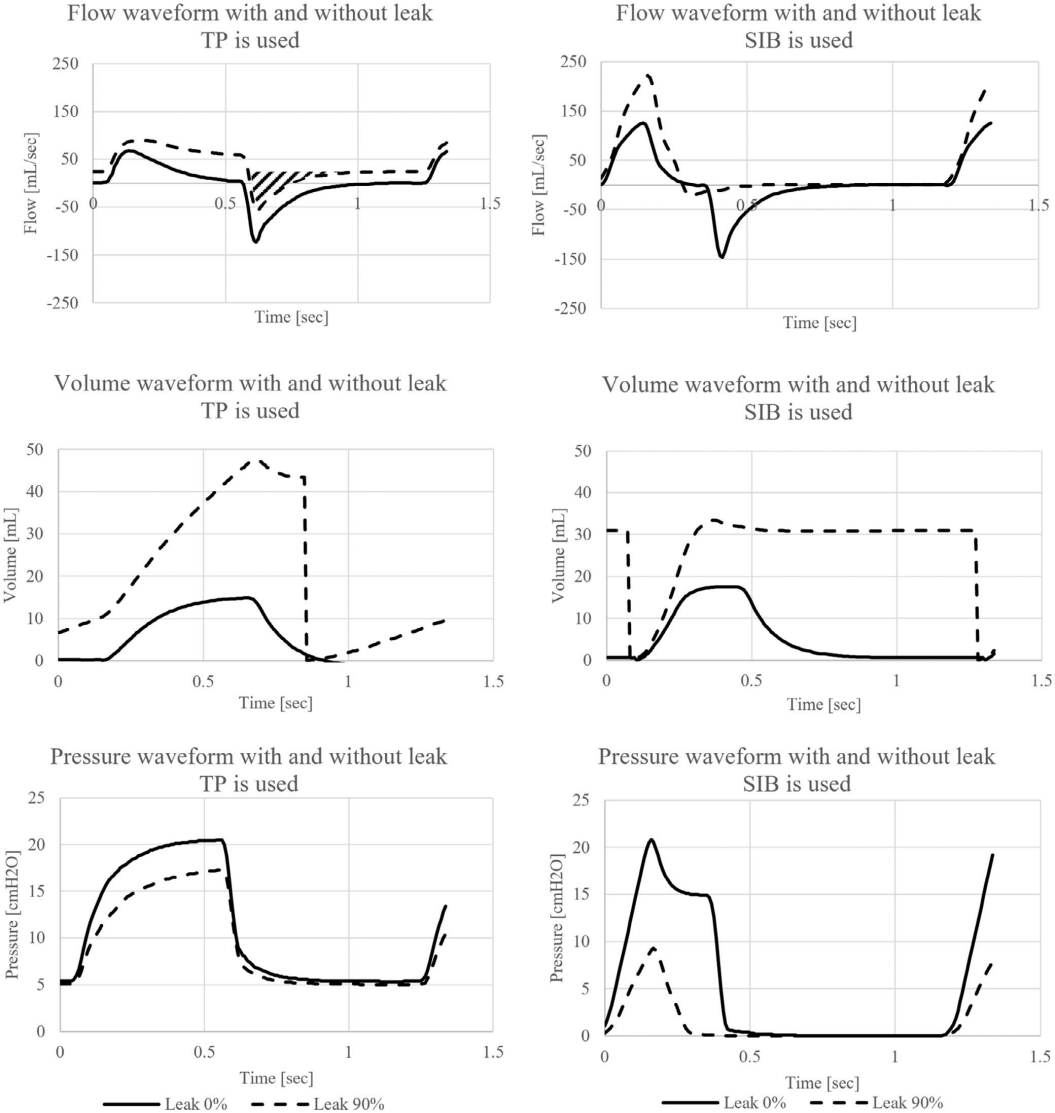
Volume, pressure, and flow waveforms for each PPV device with and without mask leaks. The flow waveform of the T‐piece resuscitator showed a positive offset at the baseline during mask leaks, resulting in a more significant error than SIB.

The waveforms of flow, volume, and airway pressure for both 0% and 90% mask leaks are depicted in Figure 3 for each device. The TP resuscitator flow waveform exhibited a positive offset at the baseline when mask leaks occurred. The tidal volume, calculated as the integral of expiratory flow, indicated that the tidal volume measured in the test lung for a 90% leakage was 12 mL, whereas the tidal volume measured at the mask was 4 mL. The compensated tidal volume measured at the mask was 10 mL after adding the offset (hatched area). By contrast, the SIB demonstrated the tidal volumes of 5 mL at the test lung and 2 mL measured at the mask unaffected by the offset.

Analysis of the airway pressure waveforms revealed that the TP resuscitator gradually reached 20 cmH_2_O slowly, whereas the SIB reached 20 cmH_2_O more rapidly, suggesting that the TP resuscitator imposed a lower lung load.

## DISCUSSION

This comprehensive study thoroughly examined the correlation between tidal volume and airway pressure in both test lungs and masks. The investigation involved manipulating the percentage of mask leak volume using an experimental system, which included a test lung, an automated bag compression machine, and two blocks to secure a mask while controlling the mask leaks.

The study revealed that (1) mask leaks resulted in an underestimation of tidal volume, with this underestimation increasing as the percentage of mask leakages increased; (2) a PPV device using a compressed gas source caused less reduction in tidal volume delivered to the lung compared with a PPV device without a compressed gas source. Additionally, PPV devices without a compressed gas source were more likely to decrease the tidal volume compared with those with a compressed gas source. Although the ventilation rate did not have a significant influence, high ventilation rates were prone to errors, even with small leaks.

Currently, no consensus exists regarding the appropriate tidal volume or the acceptable percentage of mask leaks. Considering that an excessive tidal volume may cause lung injury,^12^, ^13^ accurate tidal volume measurement becomes crucial. O'Donnell et al. reported a relationship between the tidal volume at the mask and that at the test lung during mask leaks.^8^ Øystein et al. demonstrated that mask leaks of up to 80% did not compromise the recommended pressure and volume.^14^ Our study further indicates that more significant mask leaks result in a quantitative underestimation of the tidal volume. This suggests that the tidal volume measurement at the mask depends on the percentage of mask leaks and the specific PPV device used. In PPV devices that require a compressed gas source, mask leaks do not significantly reduce the tidal volume. Unlike devices without a compressed gas source, flow waveforms indicated that devices with a compressed gas source maintained a baseline flow that counteracted the expired flow from the test lung. A smaller tidal volume displayed on the RFM, particularly with a compressed gas source, may prompt healthcare providers to deliver a higher tidal volume. However, our results suggest that such an attempt may lead to excessive tidal volume if mask leaks are present, due to underreporting of the tidal volume. To prevent the delivery of excessive tidal volume, monitoring the tidal volume while considering the percentage of mask leaks proves essential. Reducing mask leakage allows for more accurate tidal volume monitoring.

The accurate measurement of the tidal volume in the presence of mask leaks presents a significant future challenge. This study demonstrated the potential for compensating tidal volume by adjusting the baseline flow. However, determining the baseline value during the procedure remains an unresolved issue. Additionally, other contributing factors, aside from the baseline offset, were observed, and addressing these factors presents another future challenge. We also emphasized the significance of mask leaks; however, confirming PIP, PEEP, V_TE_, VR, and %leak simultaneously during PPV remains difficult. However, as V_TE_ is affected by mask leaks, monitoring V_TE_ and %leak could be beneficial in distinguishing the difference between PPV devices.

However, this study assumed that bagging and mask leaks remained constant for quantitative evaluation, presenting a limitation.

## CONCLUSION

This study systematically investigated the effect of mask leaks on tidal volume and airway pressure, emphasizing significant differences between measurements at the mask and the lung. It underscores the importance of precise tidal volume measurement and monitoring, particularly in the presence of mask leaks, to prevent potential lung injury caused by excessive tidal volume. Additionally, it was observed that PPV devices utilizing compressed air sources exhibited greater variability. Monitoring tidal volume and accounting for the percentage of mask leaks is essential to avoid delivering excessive tidal volume. Accurately measuring tidal volume in the presence of mask leaks presents a significant challenge for the future.

## AUTHOR CONTRIBUTIONS

*Conceptualization*: Fumihiko Takatori, Tetsuya Isayama, Shigeharu Hosono, and Kiyotaka Iwasaki. *Methodology*: Fumihiko Takatori and Tetsuya Isayama. Formal analysis: Fumihiko Takatori, Tetsuya Isayama, and Shigeharu Hosono. *Writing—original draft preparation*: Fumihiko Takatori. –Writing—review and editing: Fumihiko Takatori, Tetsuya Isayama, Shigeharu Hosono, and Kiyotaka Iwasaki. All authors have read and agreed to the published version of the manuscript.

## CONFLICT OF INTEREST STATEMENT

Fumihiko Takatori (First author) is an employee of Nihon Kohden. No financial support for this study was received from Nihon Kohden.

## ETHICAL APPROVAL

This article does not involve any studies with human participants conducted by the authors.

## Data Availability

Author elects to not share data.
